# Cadmium removal from aqueous solution by green synthesis iron oxide nanoparticles with tangerine peel extract

**DOI:** 10.1186/s40201-015-0237-4

**Published:** 2015-12-16

**Authors:** Mohammad Hassan Ehrampoush, Mohammad Miria, Mohammad Hossien Salmani, Amir Hossein Mahvi

**Affiliations:** Department of Environmental Health, School of Public Health, Shahid Sadoughi University of Medical Science, Yazd, Iran; Center for solid Waste Research, Institute for Environmental Research, Tehran University of Medical Science, Tehran, Iran

**Keywords:** Iron oxide nanoparticles, Cadmium removal, Tangerine peel extract

## Abstract

**Background:**

The adsorption process by metal oxide nanoparticles has been investigated an effective agent for removing organic and inorganic contaminants from water and wastewater. In this study, iron oxide nanoparticles were synthesized in the presence of tangerine peel extract as adsorbent for cadmium ions removal from contaminated solution. Iron oxide nanoparticles prepared by co-precipitation method and tangerine peel extract was used to prevent accumulation and reduce the diameter of the particles. Effect of various parameters such as contact time, pH, metal concentration and adsorbent dosage was determined on the removal efficiency.

**Results:**

The different concentrations of tangerine peel had an impact on the size of nanoparticles. As, increasing the concentration of tangerine peel extract from 2 to 6 % the average size of synthesized iron oxide nanoparticles decreased 200 nm to 50 nm. The maximum removal of cadmium ions (90 %) occurred at pH of 4 and adsorbent dose of 0.4 g/100 ml. Adsorption of cadmium ions by synthesized iron oxide nanoparticles followed Freundlich adsorption model and pseudo-second-order equation.

**Conclusion:**

The cadmium ions are usually soluble in acidic pH and the maximum removal of cadmium by green synthesis iron oxide nanoparticles was obtained in the pH of 4, so these nanoparticles can be a good adsorbent for the removal of cadmium from wastewater.

## Background

Cadmium usually is a trace ion in the ground water and surface water. It may be hydrated ions, complexes - such as inorganic carbonate, hydroxide, chloride, sulfate, or as organic complexes with humic acids in water [1]. The high levels of cadmium in water may be existed in the plating and coating of pipes and fittings, soldering with silver-coated tubes places [2]. Cadmium is toxic for animals and human, and recently, much attention has been on the long-term risk factors for general population health that are affected by cadmium. Cadmium can be introduced to body via inhalation, ingestion or absorption through the skin and affects on the body organs. The effects of short-term exposure on cadmium include: nausea, vomiting, diarrhea, muscle cramps, dry mouth, impaired senses, liver damage, convulsions, shock and renal defects, influences on the lung and the cardiovascular system, liver, and nervous system [3, 4]. Long-term effects of exposure on cadmium include the impacts on the lungs, kidneys, bones, growth and carcinogenic. Plants, fruits and vegetables are grown in the fertile soil with superphosphate fertilizer relation to the soil has higher levels of cadmium. The daily intake cadmium for the average person weighing 70 kg was estimated 25–60 μg by The United States of America and Europe [5]. The WHO is recommended maximum allow of 10 μg/L cadmium ion in drinking water sources [6]. Therefore, in recent years, considerable attention has been devoted to the study of effective methods to remove low concentrations of cadmium in contaminated water.

When cadmium concentration in contaminated water is less than 0.5 mg/L, treatment of water by conventional methods such as precipitation with lime and treatment with alum and ferric sulfate is not suitable to remove it and also is less effective [7]. Most researchers has proposed ion exchange, reverse osmosis, coagulation-filtration and adsorption processes as the best available technology for effective removal of cadmium to the 0.005 mg/L or 5 ppb [3, 8]. However, most of these methods have disadvantages such as expensive devices, production of toxic sludge or other waste materials, space and high energy requirements. Among these methods, adsorption has attracted the attention of many researches because it is a simple, low-cost, and effective for the removal of heavy metal ions in low and medium concentrations [9]. A number of minerals, clays, and waste materials were regularly used to remove heavy metals from water and wastewater [10]. Recently, it emphasized that the nanoparticles and nanostructure sorbents can be used as an efficient and convenient replacement instead of conventional adsorbents [11, 12]. Adsorption of metal ions on iron nanoparticles is an environmentally friendly technology that it has been studied as an effective agent for removing organic contaminants and heavy metal ions from water and wastewater [13–21].

Number various methods studied to synthesis of nanoparticles such as: co-precipitation, sol-gel synthesis, micro emulsion, and the oxidation of nanoparticles [22]. The chemical co-precipitation of ferric and ferrous ions in the alkaline solution is a commonly and large scale amount for synthesis of iron oxide nanoparticles [23]. During the separation of particles from bulk of solution, nanoparticle will lead to aggregation under Vander Waals force. The synthesis nanoparticles to agglomeration which converts a greater particles size in diameter. For prevention of agglomeration, it often used a surface modification using inorganic or organic material [24]. Most of water soluble polymers or surfactants can often be used a good dispersion or capping agent, but they are toxic for environment. In this study, tangerine peel extract a green material was applied to synthesis of iron oxide nanoparticles by co-precipitation methods and then these nanoparticles were used to remove cadmium ions from aqueous solutions. This method dose not required organic solvent for prevention of agglomeration that nanoparticles with an average diameter size of 50 nm can be produced in an aqueous peel extract solution. Also, this approach is entirely friendly environment and is not an environment pollutant.

## Methods

### Tangerine Peel Extract

Tangerine peel was collected from the local market at Yazd city. These washed with distillated water to clean the surface pollutants and dried in the air and absence of sunlight at the Lab. temperature (27 ± 2 °C). The dried peels were milled by electric milling and sieved to powders. 50 g of powders were introduced to 500 ml of distilled water in the beaker and heated to 80 °C for 15 min. After cooling to Lab. temperature filtered with 0.45 μ filter paper. This solution as capping agent was maintained in the refrigerator for further use.

### Preparation of nanoparticles

The co-precipitation method was used to prepare nanoparticles [25, 26]. For this purpose, 5.35 g of FeCl_3_ and 8.10 g of FeCl_2_.4H_2_O were separately dissolved in 500 ml of different concentration of extract peel to form a solution with the concentration of 0.1 M for Fe(III) and Fe(II) in presence of extract peel as surfactant and stabilizer. Then two solutions were mixed a ratio of 40–60 ml and raised it to 80 °C using an electric heater. Finally, NH_4_OH solution 25 % was dropped to this solution with extremely string at least 20 min until the final pH was came over than 9. The brown precipitate collected by magnetic piece and washed three times with distilled water. The treatment precipitant was dried at Lab. temperature at 1 week. Scanning electron microscopy (SEM) and dynamic light scattering (DLS) were used to specify nanoparticles size. These iron oxide nanoparticles were used to study of removal cadmium ions from aqueous solutions.

### Adsorption process

To obtain different concentrations of working and standard solution of cadmium ions, the stock solution of 1 g/l of cadmium ions was prepared with pure cadmium nitrate Cd (NO_3_)_2_.4H_2_O and distilled water. Experiments were performed on four periods (15, 30, 60, 90 min), three levels of pH (2, 4, 6.5), four different concentrations of cadmium ions (5, 10, 15, 20 mg/l), and three levels of absorbent dose (0.2, 0.4, 0.6 gr/100 ml). Then cadmium ions solution contacted with the adsorbent, shake at specified time on the 120 rpm orbital shaker, separated by a piece of magnet, and centrifuged in the 5000 rpm. In the end of each experiment, the residual cadmium concentration in solution was determinate.

### Data analysis

The remaining concentration of cadmium in clear solution was determined using of atomic absorption spectrometer (spectra model AA-20). For this purpose, different concentration (0.0, 2.0, 5.0, 8.0 and 10.0 mg/l) standard solutions of Cd^2+^ were prepared from stock solution of 100 mg/l and used for calibration of AA. Absorbance was recorded three fold at wavelength 228.8 nm and 0.5 nm in a standard mode and concentration of cadmium was calculated from Beer-Lambert equation. The removal efficiency of nanoparticles and adsorption capacity was calculated from experimental data as eqs. (1 and 2).

1 $$ \%\ \mathrm{R}\mathrm{E}=\frac{\left({\mathrm{C}}_0-\mathrm{C}\right)}{{\mathrm{C}}_0}\times 100 $$

2 $$ {q}_e=\frac{\left({\mathrm{C}}_0-\mathrm{C}\right)}{\mathrm{m}}\times \mathrm{V} $$

After determining of adsorption data, using linear equations related to isotherm and kinetic models, modeling of Cd^2+^ adsorption study on the synthesized iron oxide nanoparticles were determined.

## Results and Discussion

### Characterization of synthesized iron oxide nanoparticles

**Fig. 1:**
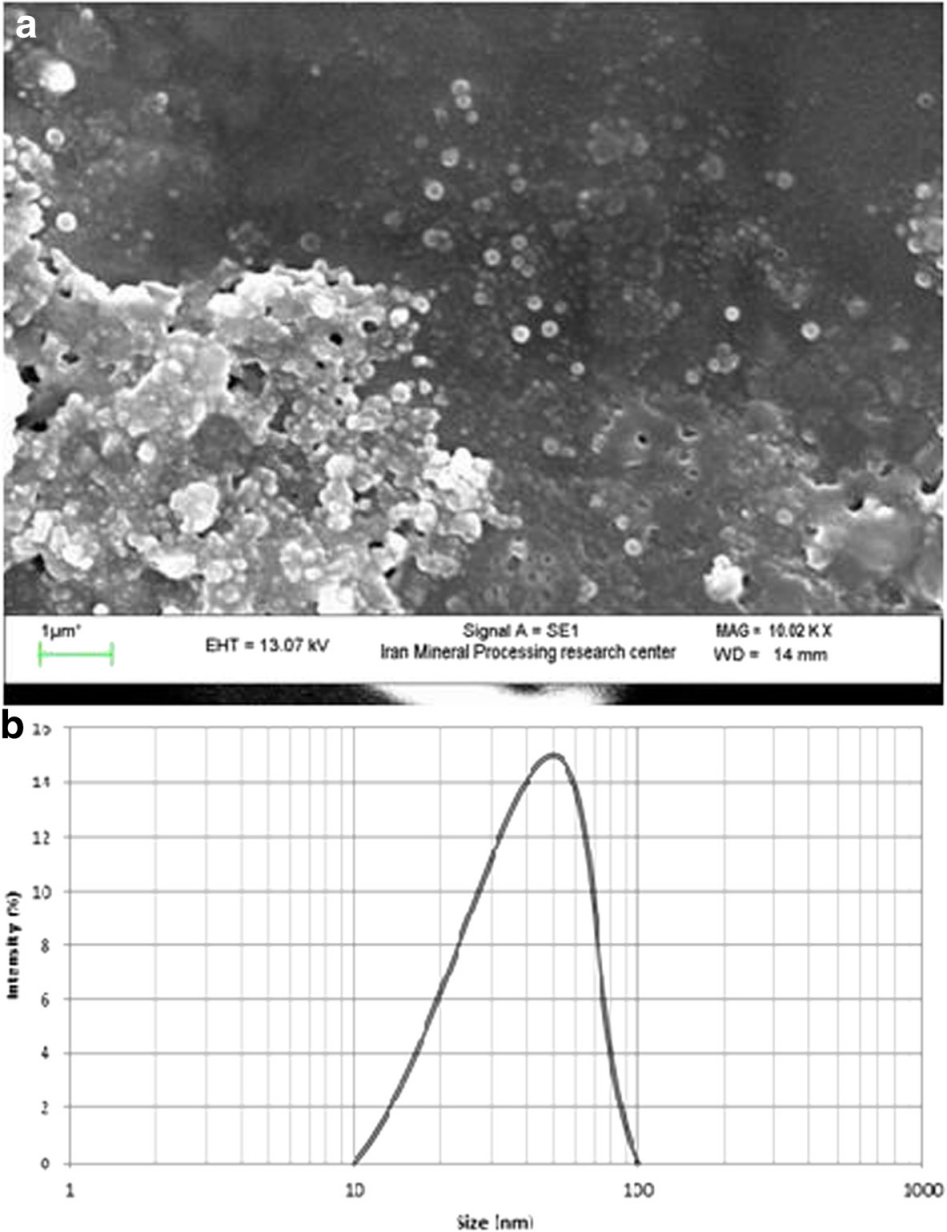
Characterization of synthesized iron oxide nanoparticles **a**) SEM image **b**) Size distribution by DLS

Iron oxide nanoparticles were prepared in the presence of various concentrations of tangerine peel extract (2, 4, 6, 8, and 10 %) that the size of them was controlled using scanning electron microscopy (SEM) and also the range size of produced particles was determined by dynamic light scattering (DLS). Average size of nanoparticles was about 200 nm at 2 % concentration of tangerine peel extract. As well as, the same average was obtained when tangerine peel extract increased to concentration of 4 %. While the tangerine peel extract reached to a concentration of 6 %, a significant decrease in the size of the iron oxide nanoparticles were appeared, so an average size was about 50 nm. Tangerine peel extract concentration of 10 % indicates severe aggregation and significant increasing in the size of the nanoparticles that the average size of nanoparticle was reached to 1 μ. Figure 1a, b shows the SEM and DLS of prepared nanoparticles at optimal conditions that was 6 % of tangerine peel extract.

Tangerine peel extract was used to prepare iron oxide nanoparticles as well as to reduce the impact on average nanoparticles size for increasing of its removal efficiency. The results showed that the concentration of tangerine peel extract can affect the nanoparticles size. So that with increasing concentration of tangerine peel extract from 2 to 6 %, the average size of nanoparticle reach out about 200 nm to 50 nm and the increasing concentration of tangerine peel extract continued up to 10 % the size of nanoparticles rose again due to the strong adhesion of nanoparticles. The tangerine peel extract concentration of 6 % is optimal for the production of iron oxide nanoparticles. Over the last few years, several synthetic methods have been focused on produce of controlled size nanoparticles and enhance to produce regular shape [27]. From SEM image predicted that iron oxide nanoparticles synthesized by tangerine peel extract of 6 % yield relatively uniform spherical shape in average range 50 nm that calculated from DLS micrograph. So, it seems that different agglomeration can occur depending on the used concentration of peel extract, which directly influences the size of particles.

Also, the synthesized iron oxide nanoparticles in presence of tangerine peel extract applied to remove of Cd^2+^ in aqueous solution. For obtaining the best condition, several experiments were done by variation of effective parameters such as contact time, pH, initial concentration and adsorbent dosage in a batch system.

### The effect of contact time

**Fig. 2:**
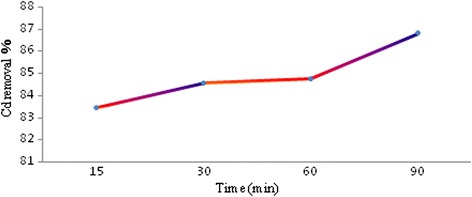
The effect of contact time on the Cd removal efficiency

The effect of contact time on cadmium removal efficiency by iron oxide nanoparticles synthesized in the presence of tangerine peel extract was investigated in an initial concentration of 5 mg/l of cadmium ions at 15, 30, 60 and 90 min. During this phase, adsorbent mass was 0.4 g/100 ml and the solution pH adjusted to 4. The effect of contact time is shown in Fig. 2. As observed, the removal rate increases with time so the optimal time is about 90 min for the best removal and this time was chosen to subsequent experiments.

According to Fig. 2, with increasing contact time, cadmium ion removal efficiency increases, because of cadmium ions are more opportunities for contact with the adsorbent surface when time increases. As can be seen from Fig. 2, the rate of cadmium ions removal was fast in the beginning times (first 15 min) due to the larger surface area of the adsorbent available. As time increases to 90 min, more amount of cadmium ions adsorb onto the surface of the adsorbent by attraction forces and cause a complete removal of cadmium ions. Therefor, the equilibrium time for this absorbent is about 90 min. Kosa et al. (2012) have been studied as heavy metals removal using carbon nanotubes modified with 8-hydroxyl-quinoline. Their results showed that removal efficiency was increased by enhancing the contact time between the adsorbent and cadmium ions. The maximum absorption occurred in the first 10 min that the results of this study are in line with the present study results [28].

### The effect of solution pH on Cd^2+^ removal

**Fig. 3:**
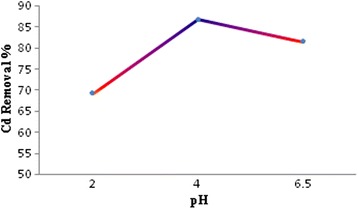
The effect of pH on the Cd removal efficiency

pH is the most important factor in the absorption process. In this study, the effect of pH on cadmium removal efficiency by prepared iron oxide nanoparticles was determined in the absorption process at pH 2, 4, and 6.5. For each experiment, the 0.4 g of adsorbent was added into the 100 ml initial cadmium concentration of 5 mg/l at each above adjusted initial pH. After a contact time of 90 min, residual concentrations of cadmium ions in the solution were measured and the removal efficiency was calculated based on the results. The results are presented in Fig. 3. The optimum pH for removal efficiency of cadmium was 4 that were used in the following experiments.

Figure 3 revealed that the removal efficiency is lower at high acidic pH. It seems that the positive charge on the adsorbent is created in the acidic pH. So, there is an electrostatic repulsion between the adsorbent and cadmium ions in solution. The hydrogen ions instead of cadmium ions are placed in to the adsorbent sites when the amount of hydrogen ions increases in solution, and so the removal efficiency decreases. This study showed that changing of pH from 2 to 4, the removal efficiency increases but it has a decreasing trend. The similar observation was obtained in our previous study [29] . The zinc oxide nanoparticles were used for the removal of cadmium at high ionic strength solution that with a change in pH from 4 to 5, the removal efficiency of cadmium increased in a great deal, and when the pH increased to 7, the removal efficiency of cadmium has had a gradual increase. Thus, the highest removal efficiency gained in pH = 7 that was 89.6 %, and the lowest efficiency measured as 38 % in pH = 4.

A study conducted by Afkhami et al. (2010), The alumina nanoparticles modified with 2, 4-di-nitro phenyl hydrazine was used to remove heavy metals such as Pb (II), Cd (II), Cr (III), Co (II), Ni (II) and Mn (II). The specific effect of pH was determined to change of pH 1.5–5.5 ranges. The results showed that removal efficiency increases up to pH of 5 and then decreases with excess of pH up to 5. These results are in line with the present study [30]. The obtained results of the present study are in line with the mentioned research findings.

### The effect of initial concentration on Cd^2+^ removal

**Fig. 4:**
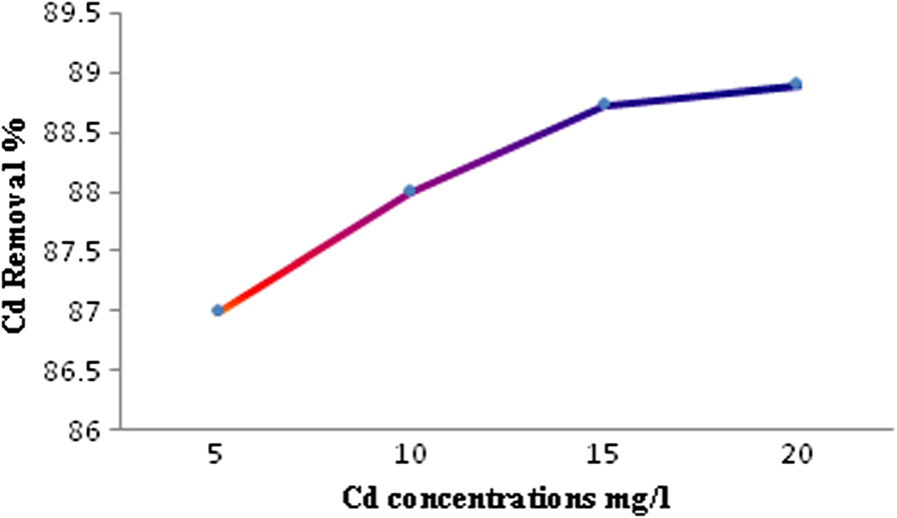
The effect of initial concentration on the Cd removal efficiency

Effect of initial cadmium concentration on the removal efficiency was investigated in the pH = 4, the mass of nanoparticles 0.4 g/100 ml solution with a concentration 5, 10, 15, and 20 mg/L of cadmium ions. The results are shown in Fig. 4. As observed, with increasing cadmium concentration of 5–20 mg/L, the removal efficiency is increased from 87 to 88.7 %.

An increased ratio of initial number of cadmium ions to the available surface area resulted in high concentration; hence fractional adsorption dependent on initial concentration. For a given adsorbent dose the total number of available adsorption active sites is constant thereby adsorbing almost the same amount of adsorbate, thus a decrease in the removal of adsorbate was resulted to an increase in initial concentration of cadmium. Similar results were also reported [31].

### The effect of adsorbent mass on Cd^2+^ removal

**Fig. 5:**
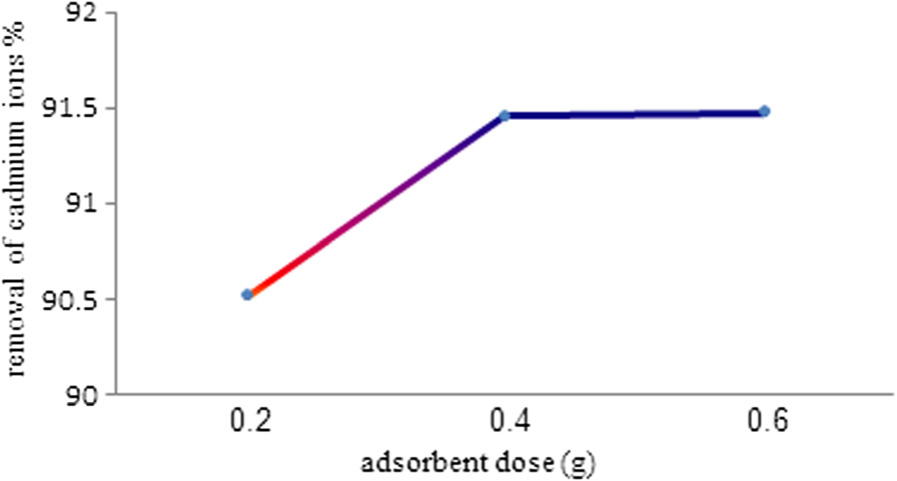
The effect of adsorbent mass on the Cd removal efficiency

In this study, the effect of iron oxide nanoparticles mass on the removal of cadmium was examined by changing of the mass of 0.2, 0.4, and 0.6 g at the 100 ml initial cadmium concentration of 15 mg/l. All suspensions were shacked 90 min, separated of adsorbent, measured the residual cadmium ions in solution and calculated the removal efficiency. The obtained results are presented in Fig. 5. The results showed the increasing of adsorbent mass from 0.2 to 0.6 g/ 100 ml the removal efficiency increased up to mass of 0.4 g and then remained constant. Hence value of 0.4 g/100 ml of adsorbent dosage was conducted as optimum value.

The influence of adsorbent dose on adsorption of cadmium ions was studied for obtaining the right adsorbent mass. Figure 5 shows, at the constant cadmium ions concentration, the adsorption percent of cadmium ions increases with increasing mass of the adsorbents. This is due to the existence of more available sites on surface of adsorbents at higher doses. When adsorbent dose was increased from 0.4 to 0.6 % w/v, the removal of cadmium ions had a low increasing. This effect may be due to the fact that some adsorption sites remain unsaturated during the batch adsorption process. This drop in adsorbed amount per unit mass of adsorbent is a routine behavior in the batch process.

### Kinetic and isotherm adsorption

The adsorption data are usually described by adsorption equilibrium isotherms that indicate the effect of the initial concentration of pollutant on the adsorption quantity. In present study, the equilibrium adsorption of cadmium by iron oxide nanoparticles was explored at the contact time of 90 min., the adsorbent dose of 0.4 g/100 ml, cadmium initial concentrations in range of 5, 10, 15, 20 mg/L and the temperature of 25 °C. The *Langmuir* and *Freundlich* models were applied to determine the adsorption isotherm for cadmium removal by synthesized iron nanoparticles that the obtained results are presented in Table 1.

**Table 1:** Linear equation and adsorption isotherm parameters for Cd^2+^ removal

Isotherm Linear equation	Langmuir			Freundlich		
	$\frac{1}{{\mathrm{q}}_{\mathrm{e}}}=\frac{1}{{\mathrm{q}}_{\max }\ {\mathrm{K}}_{\mathrm{l}}{\mathrm{C}}_{\mathrm{e}}}+\frac{1}{{\mathrm{q}}_{\max }}$			$\ln\ {\mathrm{q}}_{\mathrm{e}} = \ln {\mathrm{K}}_{\mathrm{F}} + \frac{1}{\mathrm{n}} \ln\ {\mathrm{C}}_{\mathrm{e}}$		
Parameters	R^2^	b	q _max_	R^2^	n	k_F_
Obtained values	0.993	0.117	15.5	0.997	0.866	1.789

One of the most salient factors to design an adsorption system (to determine the optimum contact time) is anticipating the speed of the adsorption process that is controlled by isotherm and kinetic system. The common isotherm models are Freundlich and Langmuir that the linear equations are presented in 1. According to regression coefficient (Table 1), experiments data of cadmium adsorption on synthesized iron oxide nanoparticles followed Freundlich models better than those for the Langmuir model. Freundlich equation is an empirical adsorption isotherm equation that can be applied in case of low and intermediate concentration ranges. Freundlich isotherm gives the parameters, n, indicative of bond energies between metal ion and the adsorbent and K_F_, related to bond strength [32]. The slope n and K_F_ from the linear equation was 0.866 and 1.789, respectively, satisfying of the condition for favorable adsorption.

In order to ascertain cadmium adsorption kinetic on iron oxide nanoparticles, the experiments were carried out under the optimized conditions and four different contact times as 15, 30, 60, and 90 min that were selected to obtain adsorption kinetic. *Pseudo-first order* and *pseudo- second order* equation were considered to determine the adsorption kinetic. The obtained results are presented in Table 2.

**Table 2:** Kinetic equation and obtained results for Cd^2+^ removal

	*pseudo-first order*		*pseudo*-*second order*	
model	$\log \left({q}_e-{q}_t\right)= \log {q}_e-\frac{k_1}{2.303}t$		$\frac{t}{q_t}=\frac{1}{k_2{q}_e^2}+\frac{1}{q_e}t$	
Equation	Y = −0.0352x-2.3298	R^2^ = 0.798	Y = 0.9163 x + 0.9007	R^2^ = 0.999
Obtained Value	K_1_ = 0.081	q_e_ = 4.7	K_2_ = 1.119	q_e_ = 10.9

The pseudo-first-order and pseudo-second-order equations were applied to assess the suitability of the rate equation for the experiment data. The obtained rate equations were compiled in Table 2. The results show that the correlation coefficients for second order rate equations (0.999) were higher than those for the first order rate equations. Hence, the pseudo second order rate equation is more suitable to explain the cadmium adsorption on the iron oxide nanoparticles. In case of pseudo second order kinetics, the plot of (t/q_t_) versus t gives a linear relationship that allows computation of q_e_ and K_2_. The second order rate constant (k_2_) value and equilibrium adsorption capacity (q_e_) was 1.119 g.mg^−1^.min^−1^ and 10.9 mg.g^−1^ for cadmium adsorption by synthesized iron nanoparticles in presence of tangerine peel extract.

## Conclusion

The use of peel extracts as stabilizer agent for preparation of metal oxide nanoparticles is inexpensive, and eco-friendly. It is especially for preparation of nanoparticles that have been free of toxic contaminations. The peel extract can controlled the size and morphology of nanoparticles during synthesis process. Simple green synthesis of iron oxide nanoparticles through co-precipitation in alkali condition can exhibit an excellent adsorption for the cadmium ions that followed Freundlich adsorption model and pseudo-second-order equation. The obtained results of this investigation indicated that the synthesized adsorbent was more able to remove cadmium ions.
